# Association of imaging abnormalities of the subcallosal septal area with Alzheimer's disease and mild cognitive impairment

**DOI:** 10.1016/j.crad.2017.04.021

**Published:** 2017-11

**Authors:** C.L. Gan, M.J. O'Sullivan, C. Metzler-Baddeley, S. Halpin

**Affiliations:** a Department of Neuroradiology, University Hospital of Wales, Cardiff, UK; b Cardiff University Brain Research Imaging Centre, School of Psychology, Cardiff, UK; c Department of Clinical Neuroscience, Institute of Psychiatry, King's College London, London, UK

## Abstract

**Aim:**

To evaluate the use the distance between the adjacent septal nuclei as a surrogate marker of septal area atrophy seen in Alzheimer's disease (AD).

**Materials & Methods:**

Interseptal distance (ISD) was measured, blind to clinical details, in 250 patients who underwent computed tomography (CT) of the brain at University Hospital of Wales. Clinical details including memory problem history were retrieved. An ISD cut-off value that discriminated those with and without memory symptoms was sought. ISD measurements were also made in 20 AD patients. To test both the method and the defined cut-off, measurements were then made in an independent cohort of 21 mild cognitive impairment (MCI) patients and 45 age-matched healthy controls, in a randomised and blinded fashion.

**Results:**

ISD measurement was achieved in all patients. In 28 patients with memory symptoms, the mean ISD was 5.9 mm compared with 2.3 mm in those without overt symptoms (*p=*0.001). The optimum ISD cut-off value was 4 mm (sensitivity 85.7% and specificity 85.8%). All AD patients had an ISD of >4 mm (mean ISD= 6.1 mm). The mean ISD for MCI patients was 3.84 mm compared with 2.18 mm in age-matched healthy controls (*p=*0.001). Using a 4 mm cut-off correctly categorised 10 mild cognitive impairment patients (47.6%) and 38 healthy controls (84.4%).

**Conclusion:**

ISD is a simple and reliable surrogate measurement for septal area atrophy, applicable to CT and magnetic resonance imaging (MRI). It can be used to help select patients for further investigation.

## Introduction

Alzheimer's disease (AD) is the commonest cause of dementia and it affects around 5.4 million people in the US.^1^ Patients typically present with progressive memory impairment and involvement of other cognitive domains or skills, which impair social function and activities of daily living.^2^ The diagnosis of AD is often difficult especially near the onset of symptoms. Recent diagnostic guidelines have attempted to integrate neuroimaging and other biomarkers. The majority of the neuroimaging research in AD has centred around mesial temporal lobe atrophy, which shows volume loss in hippocampi, amygdala, entorhinal cortex, and posterior cingulate cortex^3^, ^4^, ^5^, ^6^; however, none of these markers are straightforward to measure and evaluate and many are suitable only for magnetic resonance imaging (MRI). Very few studies have focused on the subcallosal grey matter in relation to AD despite the fact that atrophy of this region occurs early and is just as characteristic of AD.^7^

Although the imaging diagnosis for AD is difficult, various studies have shown that hippocampal or mesial temporal lobe atrophy can be quantified by volumetric MRI^5^, ^6^ or visual rating methods^8^, ^9^; however, volumetric MRI requires rigorous standards for image acquisition and analysis and is not suitable for routine clinical use. Thus far, visual rating methods for measurement have been found to be relatively insensitive for identifying mild AD or mild cognitive impairment (MCI) and often introduce problems of reliability especially when protocols are complex. An increased cella media index has also been described in AD patients, but is relatively non-specific and greatly influenced by the general atrophy state of the brain.^10^

**Figure 1:**
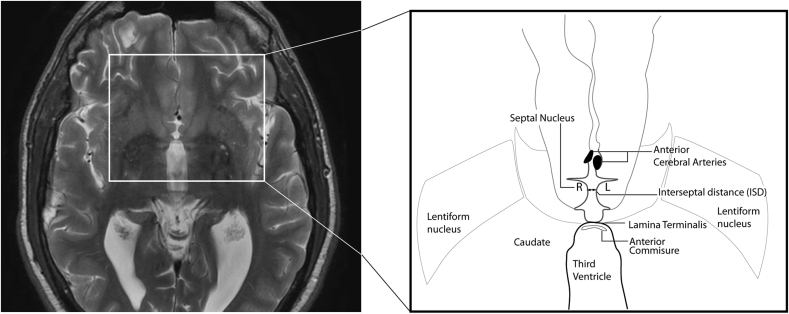
Axial T2-weighted image of an adult brain demonstrating anatomical landmarks for ISD measurement.

It has been the authors' clinical experience that atrophy of the septal nuclei can commonly be seen in conditions associated with hippocampal atrophy, principally in AD, but also following head injury or in chronic alcohol excess. It was hypothesised that a widening of the interseptal distance (ISD), defined as the minimal distance between the nuclei of each hemisphere, could act as a surrogate marker for atrophy within core regions implicated in AD and other memory disorders. This distance can be measured accurately and easily on axial computed tomography (CT) or MRI at the level of AC-PC (anterior commissure to posterior commissure) line, as the distance between the medial convexities of the septal nuclei, posterior to the anterior cerebral arteries (Fig 1). It was hypothesised that by simple measurement of the ISD, patients with memory disorders can be differentiated from the normal population, so that it can be used as a simple but useful screening tool for AD.

## Materials and methods

### Assessment of normal variation of ISD within an unselected hospital population

To observe the distribution of the ISD within a general population, the ISDs in 250 consecutive patients who had undergone CT of the brain were measured. The CT brain protocol included axial imaging using a GE 750 Discover HD (General Electric Healthcare Milwaukee, IL, USA) 64-slice multidetector CT system of 2.5 mm sections from foramen magnum to vertex (120 kV and 265 mA; 32 cm field of view [FOV]; allowing for a maximum of 2.3 noise factor; 860 mGy average dose). The CT examinations were requested either by general practitioners or hospital doctors for a variety of indications, but not specifically for the diagnosis of memory disorder. All patients' identifiable details were anonymised. Patients with intracranial mass lesions, large infarcts including the middle cerebral artery territory, and a history of neurosurgery that might affect the ISD were excluded. The measurements were done independently by an experienced neuroradiologist and a radiology trainee. Details recorded from the request forms including patient's age, any known diagnosis of AD, dementia, MCI, history of confusion or chronic memory problems as well as excess alcohol use were then recorded after blinded measurement of the ISD of these patients.

The measurements were then compared between the two for reproducibility analysis by using the analysis of variance method (ANOVA). Both inter-rater coefficient of variation and an intraclass correlation coefficient were calculated. The statistics were performed using the software, SPSS 17.0 (SPSS, Chicago, IL, USA).

Using the measurements done by the neuroradiologist, the range and mean of the ISD for patients with known AD, dementia, MCI, history of confusion, or chronic memory problems were compared with those without overt memory problems or history of excess alcohol use using independent *t*-test. Using different cut-off values for the ISD, the sensitivities and specificities were calculated and a receiver operating characteristic curve was plotted. An optimal cut-off value was then defined based on the perceived best combination of sensitivity and specificity.

### Analysis of ISD in patients with known diagnosis of AD

To test the proposed cut-off value for the ISD, the first 20 patients from a memory clinic database who had a diagnosis of AD were selected for ISD analysis. Identifiable details were removed and patients were anonymised for analysis. All patients met criteria for probable AD based on the National Institute of Neurological and Communicative Disorders and Stroke and the Alzheimer's Disease and Related Disorders Association (NINCDS-ADRDA) criteria.^11^ “Probable AD” reflects the greatest degree of diagnostic certainty that can be reached without neuropathology or biopsy. Briefly, all patients had (1) memory impairment and impairment in at least one other domain on cognitive testing; (2) significant impairment of daily activities; (3) evidence of progression over time. Recent proposed modifications based on the use of neuroimaging were not adopted.^12^ The aim was not to bias the evaluation of ISD by selecting patients based on focal atrophy elsewhere, but to test applicability in the widest sense. The Mini-Mental State Examination scores of these patients ranged from 12 to 27. In all patients, a routine CT brain was available from the standard assessment within the memory clinic. All had been reported in the normal way, noting, for example, white matter lesions and evidence of previous infarction or brain injury, but no volumetric or semi-quantitative assessments of atrophy had been attempted. The ISD for each patient was measured from the CT brain examination by an experienced neuroradiologist.

### Blinded analysis of MCI patients with age-matched healthy control participants

To assess the value of ISD measurement in MCI, as well as evaluating independence from similar measures of generalised atrophy, a set of 66 datasets taken from a research study investigating structural correlates of ageing and mild cognitive impairment was also examined. These included 21 patients with MCI, and 45 age-matched healthy controls. All were assessed by a single clinician. MCI was diagnosed according to the Petersen criteria.^13^ All had a Clinical Dementia Rating score of 0.5^14^ and memory impairment, which was confirmed by a score 1.5 standard deviations below the age-corrected population mean for the memory subscore of the Addenbroooke's Cognitive Examination.^15^ Individuals were excluded if they had a known cause of cognitive impairment other than the onset of neurodegenerative disease (e.g., previous head injury, neurological disease, alcohol or substance abuse) and were also excluded if they met diagnostic criteria for non-Alzheimer dementia (e.g., dementia with Lewy bodies or frontotemporal lobar degeneration).

Healthy control participants, with an equivalent age distribution, were recruited from the local community by advertisements posted on the internet and in family physician waiting rooms, newsletters, and mail shots. Exclusion criteria included a history of neurological and/or psychiatric disease, head injury, alcohol and/or drug abuse, memory or other cognitive decline, stroke, large artery or peripheral vascular disease, structural heart disease or heart failure, and contraindications to MRI.

All individuals gave informed consent to take part in a clinical, imaging, and cognitive study of mild cognitive impairment. Ethical approval was provided by the South East Wales Research Ethics Committee.

MR data were acquired using a 3 T GE HDx MRI system (General Electric Healthcare). T1-weighted structural MRI data were acquired using a three-dimensional (3D) fast spoiled gradient recalled (FSPGR) echo sequence, acquired with a matrix of 256×256×176, and field of view of 256×256×176 mm, resulting in isotropic (1 mm) resolution voxels. The timing parameters were TR/TE/TI = 7.9/3/450 ms, and the flip angle was 20^º^.

MRI images from all participants were reviewed and the ISDs were measured by a single experienced neuroradiologist. Patients' details including the underlying diagnosis were blinded from the assessor. The minimum anterior and posterior interhemispheric distances for each patient were also measured at the bases of the frontal lobes and the occipital lobes, respectively, as an indicator for the degree of generalised brain atrophy. The measurements for the MCI patients and healthy control participants were compared by independent *t*-test. The accuracy of ISD in identifying MCI patients was also assessed using the proposed cut-off value for ISD.

## Results

### Assessment of normal variation of ISD within an unselected hospital population

ISD measurement was achieved in all patients (median age: 63 years, age range: 1–105 years, male: female ratio (M:F) = 1:1.2). Measurements were rated as difficult in 11 patients. In six very young patients, the septal nuclei were so close to each other that ISD could not be measured accurately. The septal nuclei could not be located confidently in five patients as the anterior cerebral artery could not be separated from the nuclei. To compensate for this problem, after the independent assessment of ISD by the neuroradiologist and trainee, the images from these 11 patients were reviewed together and a reasonable ISD was agreed for each case.

The reproducibility analysis showed an inter-rater coefficient of variation of 10.5% and an intraclass correlation coefficient of 0.96 when the measurements of ISD were compared between the neuroradiologist and trainee. This shows that ISD measurement is highly reproducible between assessors irrespective of clinical experience.

**Figure 2:**
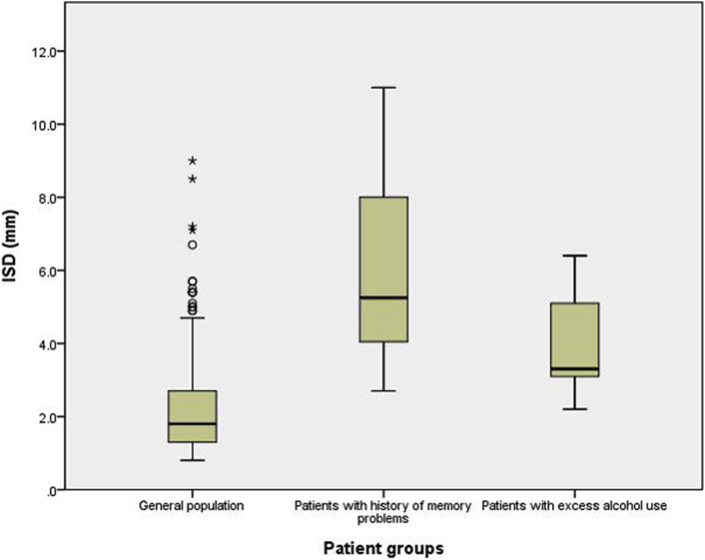
Differences of ISD between the general population and patients with memory problems and excess alcohol use.

In the original 250 ISD measurements, there were 28 patients where a known diagnosis of AD, dementia, MCI, history of confusion, or chronic memory problems had been noted on the request forms for the CT examination. The mean ISD for these patients was 5.9 mm (2.7–11 mm). There were 11 patients with a history of excess alcohol use stated on the CT brain request forms and the mean ISD for them was 4 mm (2.2–6.4 mm). The remaining 211 patients were considered a control group without overt memory disorders likely to reflect the general population. The mean ISD in this group was 2.3 mm (0.8–9 mm). There was relatively little variation in ISD with advancing age. The mean ISD for both those with noted memory difficulties, and those with noted excess alcohol use, differed from the “control” population in which neither was mentioned (*p=*0.001 for patient group with memory problems and *p=*0.002 for patient group with excess alcohol use; Fig 2).

**Figure 3:**
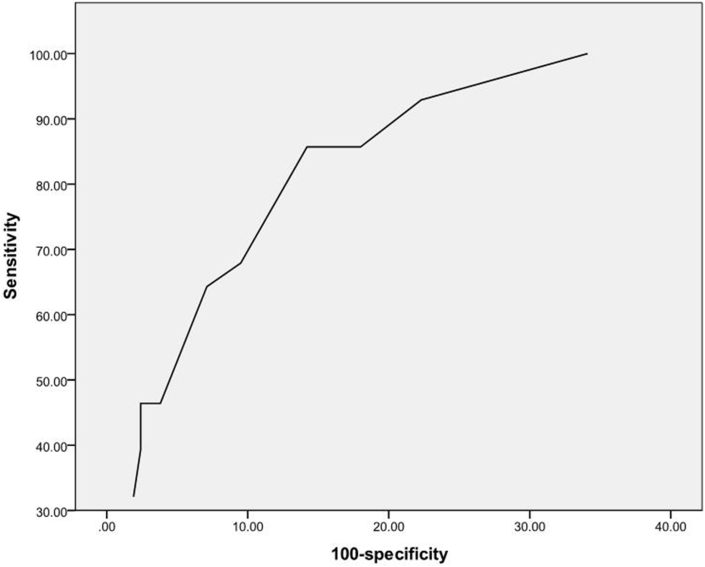
Receiver operating characteristic curve for using ISD to discriminate patients with memory problems from general population.

The receiver operating characteristic curve (Fig 3) plotted with sensitivities and specificities of using cut-off values for ISD from 2.5 to 7 mm shows that ISD measurement has a good predictive power for patients with memory problems. A ISD cut-off of 4 mm gives a sensitivity of 85.7% and a specificity of 85.7%.

### Analysis of ISD in patients with known diagnosis of AD, MCI, and age-matched healthy controls

ISD measurement was achieved in all 85 patients, including 20 AD patients (median age: 85 years, age range: 74–90 years, M:F=1:2.2), 21 MCI patients (median age: 78 years, age range: 58–90 years, M:F=1.3:1) and 45 healthy controls (median age: 69 years, age range: 53–93 years, M:F= 1.1:1). The ISD measurement was difficult in two patients where there were some movement artefacts affecting the resolution of the MRI images; however, reasonable measurements were noted for these two patients, and therefore, the results were still included in the study.

**Figure 4:**
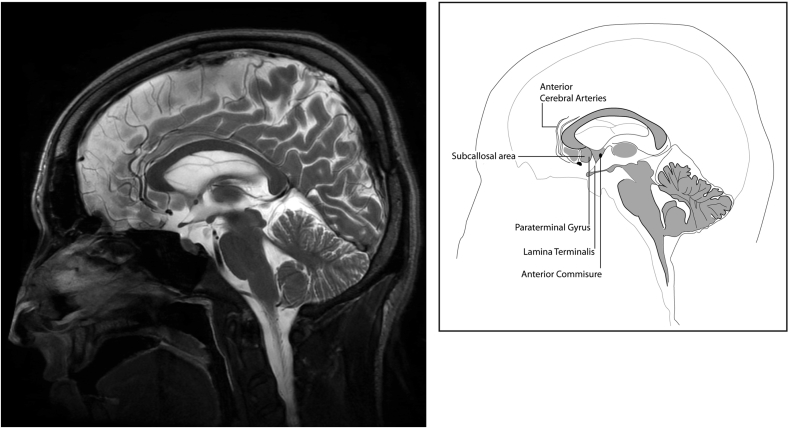
Sagittal T2-weighted image of an adult brain demonstrating the anatomy of the septal area.

The mean ISD for the different patient groups was: AD patients: 6.1 mm (4–8.1 mm); MCI patients: 3.84 mm (1–7 mm) and healthy controls: 2.18 mm (1–6.2 mm; Fig 4) The mean ISDs of both AD (based on CT) and MCI (based on MRI) groups were higher than that of the healthy control (*p*<0.001 for both comparisons).

The differences in ISD between the MCI patients and the healthy controls appear to be independent of the degree of age-related generalised atrophy of the brain as no statistically significant differences can be demonstrated on the means of anterior and posterior interhemispheric distances (Table 1). Using 4 mm as a cut-off value, 10 MCI patients and 38 healthy controls were identified correctly (sensitivity 47.6%, specificity 84.4%), whereas, all AD patients invariably had an ISD of ≥4mm.

**Table 1:** Differences in means of inter-septal, and anterior and posterior intermispheric distances.

	MCI (*n*=21)	Controls (*n*=45)	*p*-Value
Inter-septal distance (mm)	3.84±1.90	2.18±1.39	0.001
Anterior interhemispheric distance (mm)	1.25±0.42	1.10±0.22	0.66
Posterior interhemispheric distance (mm)	1.16±0.32	1.09±0.22	0.36

## Discussion

The septal area forms part of the basal forebrain cholinergic complex and is an important anterior component of the limbic system. It is located on the medial surface of the cerebral hemisphere nestled inferior to the rostrum of the corpus callosum and anterior to the third ventricle and lamina terminalis. It contains two nearly vertically oriented gyri, the subcallosal area, and the smaller paraterminal gyrus (Fig 4). The septal nuclei are subcortical grey matter nuclei deep to the septal area.^16^ Volume loss of the limbic structures and severe loss of cortical cholinergic innervation are strongly associated with AD. As the septal area is contiguous with the limbic structures, extending from the temporal lobe, volume loss of limbic structures also affects the septal area. In AD patients, the greatest percentage volumetric loss had been shown to occur in the septal area compared to other limbic structures including the hippocampus, amygdala, parahippocampal cortex, and posterior cingulate cortex.^7^ MRI-co-registered HMPAO SPET examinations of the brain have shown reduced perfusion in the septal area, as well as other limbic structures in AD patients.^17^ Septal area atrophy can be appreciated on axial CT or MRI as flattening of the normal medial convexity of the septal nuclei, which results in widening of ISD.

**Figure 5:**
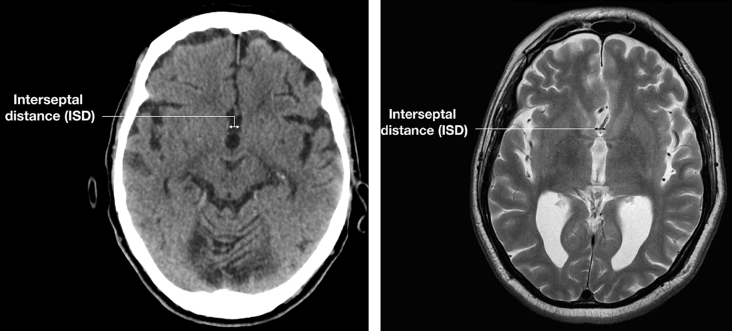
Axial CT and MRI (T2-weighted) brain images of an adult with established AD showing abnormally widened ISD.

Although measurement of the ISD is straightforward and reproducible, it does require reasonably thin axial sections on CT and MRI. On 2.5 mm sections, the cistern of the lamina terminalis posteriorly, and anterior cerebral artery branches anteriorly, and the anterior paraolfactory sulcus are identified. The septal nuclei then appear as medial convexities. In the normal patient, the CSF space connecting the cistern and the sulcus is thin, and resembles a dumbbell or narrow-stemmed wine glass (Fig 1). In the abnormal case, the CSF space is widened, in contrast to the anterior and posterior interhemispheric fissures and resembles a tumbler. With a little practice, widening of the ISD can be easily identified by both radiologists and clinicians, in routine reviewing of neuroimaging scans. Fig 5 is an example of marked ISD widening, but with closely opposed medial frontal lobes.

To the authors' knowledge, this is the first study to demonstrate a simple, quick, and reliable surrogate marker for septal area atrophy, which is a marker for the neurodegenerative states seen in AD and MCI. The results confirmed the study hypothesis that AD and MCI patients have a significantly wider ISD. This would correspond to previous studies that have shown focal atrophy of related “limbic” grey matter regions in AD patients.^3^, ^7^ Furthermore, the widened ISD is specific and is relatively independent of age-related cerebral atrophy or small vessel ischaemic damage seen in older patients.

Although the measurements were done using different imaging techniques, the ISD, in general, was wider in AD patient, than MCI patients. Therefore, by using a cut-off value of 4 mm, more AD patients than MCI patients were identified correctly. The results were similar to MRI volumetric studies showing greater hippocampal volume loss in AD patients to MCI patients.^18^, ^19^ The variation of ISD in MCI patients was probably due to the known heterogeneity of MCI. This category includes patients who progress quickly to AD, as well as others who remain stable or who have an alternative disease, which, in some cases, will spare the septal region.^20^ This is further confirmed by the greater hippocampal atrophy seen in MCI patients who progress quickly to AD than those who do not progress to AD.^21^ It is speculated that ISD may help in predicting those patients who will progress to AD.

Differential atrophy in the hippocampal subfields by using a automated segmentation software has been described in a recent study which allows better detection of MCI, compared to conventional mesiotemporal volumetry.^22^ However, in this study, we have proposed a quicker and easier quantitative method of identifying AD and MCI which can be used in routine examination, and not only in MRI but also in CT. In many healthcare settings, MRI is less easily accessible than CT, and so there are advantages in having a quick and simple method that can be equally well applied to CT and MRI.

Other measurements, such as the cella media index, have been described as being useful in AD patients. These relate the width of mesial temporal structures to the width of the whole brain, and are a relatively non-specific marker of atrophy.^10^ The ISD is more specific, being a more direct marker of atrophy of affected nuclei; however, it is interesting to speculate on exactly why the ISD is increased in AD. Without a direct volumetric study of the septal area, the increasing distance directly reflects atrophy of the septal nuclei; however, the adjacent brain is intimately involved with memory. For example, the substantia innonimata is directly adjacent to the septal nuclei, and atrophy of the substantia innominata and nucleus of Meynert has been described in AD, with an imaging correlation on MRI.^23^, ^24^ It is therefore probably an over-simplification to claim that the association of the imaging observation of a widened ISD is entirely due to loss of neurons confined to the septal nuclei. It is more likely that the observed widening reflects a somewhat more generalised regional atrophy of memory-related cholinergic neurons. The message contained within this paper is that a simple observation, made on routine CT, can identify a large percentage of patients with AD or who are at risk of developing AD. The negative finding is just as important: no patients in the present study with an ISD of <4 mm had AD.

Most patients with a large ISD (>5 mm) also had characteristic CT findings of enlarged temporal horns, and mesial temporal thinning: these patients are usually straightforward to assess by conventional imaging techniques; however, it is a common and reassuring observation in daily clinical practice to look at the ISD in patients where the mesial temporal lobes are equivocal on routine CT.

The main limitation of the present study is lack of histopathological verification. Although different studies have shown septal area atrophy in AD patients, the ISD is not a direct volumetric measurement for the septal area, but a surrogate measurement for it. The cross-sectional area of the nuclei or the height of the medial convexity could be measured directly on axial scans, but these measurements would be more complex, time-consuming, prone to interobserver error, and would convey no additional useful measurement compared to ISD measurement. Further research to correlate the ISD with hippocampal and septal area volume in AD and MCI patients has been initiated as a continuation of this study to establish their relationship and to compare superiority of tests.

The other limitation to the present study was that a full medical history for every patient was not obtained in the initial patient group to observe the variation of ISD in the general population. Memory problems may have been underdiagnosed among these patients as the authors relied solely on the information written in the request forms for the CT brain examination. The referring clinicians might not have included the relevant details of the patients' memory state and so patients with memory problems may have been included within the study's normal range. It was also assumed that patients with any chronic memory problems have a wider ISD than the normal controls; however, a major strength of this study is that this is based in a large and realistic hospital population. Furthermore, by using a cut-off ISD value suggested from the assessment of ISD in the initial patient group, patients with AD and MCI could be differentiated from healthy controls. Of course, there is some overlap between the groups, particularly between the MCI and the AD patients; however no AD patient had an ISD of <4 mm. The cut-off for ISD may be different for varying population groups, and determination of a more accurate population based cut-off value will require further studies on larger but different population groups.

ISD measurement is a cheap, simple, reliable, and easily reproducible method to measure septal area atrophy as a marker for neurodegenerative states seen in patients with memory problems. It can be used as a simple but valuable tool to differentiate patients with memory problems, in particular AD and MCI, from the normal population, with reasonable accuracy. Widening of the ISD can be easily recognised by both clinicians and radiologists, not only in MRI but also in routine CT brain scans. Use of ISD in routine reporting can act as useful screening test for AD and MCI, and help select patients for further investigations, so that diagnosis can be achieved at an earlier stage.
